# 1-[4-Bromo-2-(tri­fluoro­meth­oxy)phen­yl]-3-methyl-1*H*-1,2,4-triazole

**DOI:** 10.1107/S1600536814010083

**Published:** 2014-05-10

**Authors:** C Sandeep, Basavaraj Padmashali, P. A. Suchetan, Rashmi S. Kulkarni

**Affiliations:** a Department of Chemistry, Sahyadri Science College (Autonomous), Shimoga 577 203, Karnataka, India; b Department of Studies and Research in Chemistry, School of Basic Sciences, Rani Channamma University, Belagavi 591 156, Karnataka, India; c Department of Studies in Chemistry, U.C.S., Tumkur University, Tumkur, Karnataka 572 103, India; d PG Department of Chemistry, Jain University, Bangalore 560 019, Karnataka, India

## Abstract

In the title compound, C_10_H_7_BrF_3_N_3_O, the dihedral angle between the benzene and triazole rings is 23.17 (12)° and the C atom of the –CF_3_ group deviates from its attached ring plane by 1.147 (3) Å. In the crystal, mol­ecules are linked by C—H⋯N inter­actions, generating *C*(7) chains running along [010].

## Related literature   

For the anti­bacterial activity of 1,2,4-triazoles, see: Gabriela *et al.* (2009 ▶); Palekar *et al.* (2009 ▶). For their anti­viral activity, see: Upmanyu *et al.* (2006 ▶). For anti­microbial agents, see: Badr & Barwa (2011 ▶), and for anti­mycotic activity such as voriconazole, see: Haber (2001 ▶).
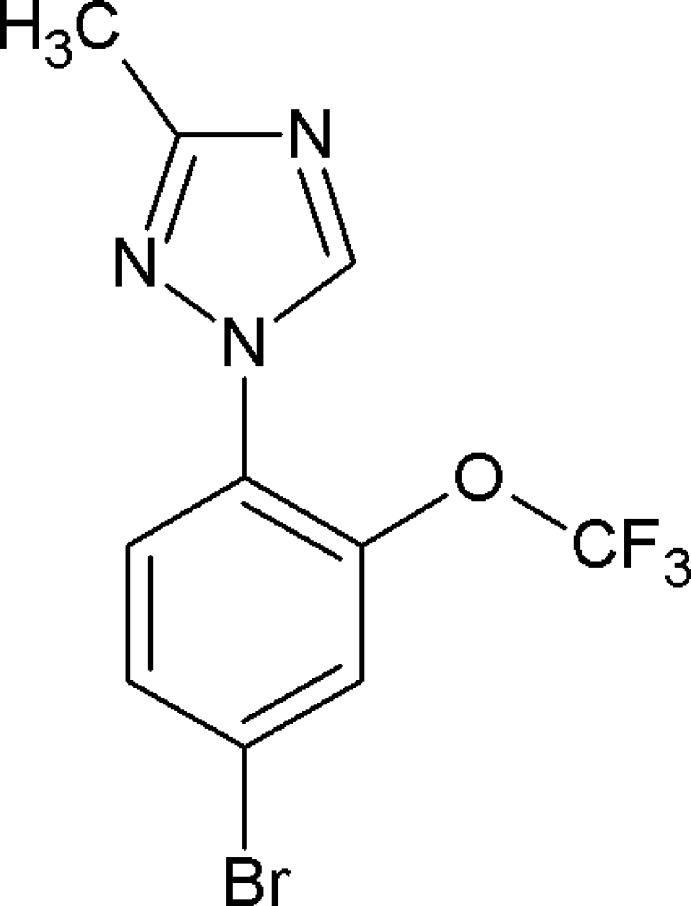


## Experimental   

### 

#### Crystal data  

C_10_H_7_BrF_3_N_3_O
*M* *_r_* = 322.10
Monoclinic, 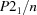

*a* = 5.2389 (3) Å
*b* = 16.1548 (8) Å
*c* = 14.0315 (7) Å
β = 92.673 (3)°
*V* = 1186.24 (11) Å^3^
*Z* = 4
Mo *K*α radiation
μ = 3.50 mm^−1^
*T* = 293 K
0.33 × 0.21 × 0.14 mm

#### Data collection  

Bruker APEXII CCD diffractometer
Absorption correction: multi-scan (*SADABS*; Bruker, 2009 ▶) *T*_min_ = 0.419, *T*_max_ = 0.613
37092 measured reflections
3499 independent reflections
2373 reflections with *I* > 2σ(*I*)
*R*_int_ = 0.051

#### Refinement  

*R*[*F* ^2^ > 2σ(*F* ^2^)] = 0.038
*wR*(*F* ^2^) = 0.135
*S* = 0.99
3499 reflections
165 parameters
H-atom parameters constrained
Δρ_max_ = 0.41 e Å^−3^
Δρ_min_ = −0.68 e Å^−3^

### 

Data collection: *APEX2* (Bruker, 2009 ▶); cell refinement: *SAINT-Plus* (Bruker, 2009 ▶); data reduction: *SAINT-Plus*; program(s) used to solve structure: *SHELXS97* (Sheldrick, 2008 ▶); program(s) used to refine structure: *SHELXL97* (Sheldrick, 2008 ▶); molecular graphics: *Mercury* (Macrae *et al.*, 2008 ▶); software used to prepare material for publication: *SHELXL97*.

## Supplementary Material

Crystal structure: contains datablock(s) I. DOI: 10.1107/S1600536814010083/hb7221sup1.cif

Structure factors: contains datablock(s) I. DOI: 10.1107/S1600536814010083/hb7221Isup2.hkl

Supporting information file. DOI: 10.1107/S1600536814010083/hb7221Isup3.cml

CCDC reference: 1000730

Additional supporting information:  crystallographic information; 3D view; checkCIF report

## Figures and Tables

**Table 1:** Hydrogen-bond geometry (Å, °)

*D*—H⋯*A*	*D*—H	H⋯*A*	*D*⋯*A*	*D*—H⋯*A*
C2—H2⋯N3^i^	0.93	2.59	3.511 (3)	169

Symmetry code: (i) 
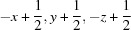
.

**Fig. 1.**
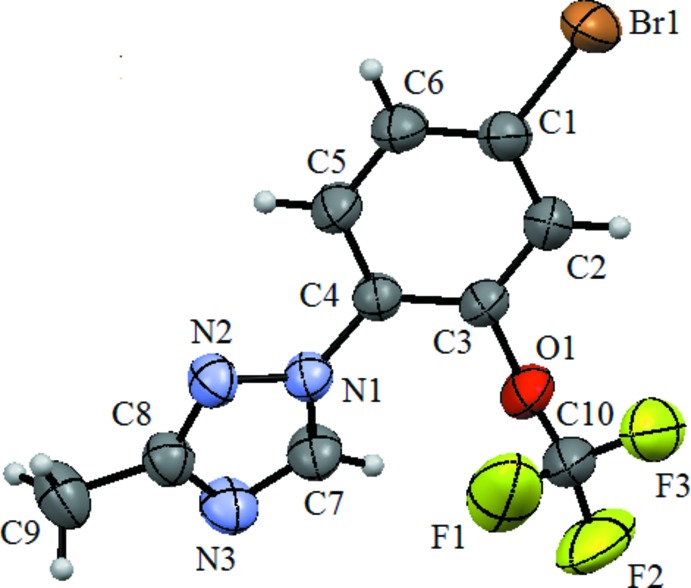
Molecular structure of the title compound, showing displacement ellipsoids drawn at the 50% probability level.

**Fig. 2.**
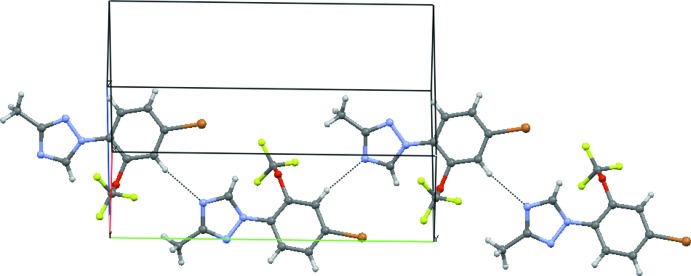
Linking of molecules in the crystal structure through C—H···N interactions into C(7) chains.

## supplementary crystallographic information

### 1. Introduction

1,2,4-triazole containing ring system have been incorporated into a wide variety of therapeutically inter­esting drug candidates including anti-inflammatory, CNS stimulants sedatives, anti­bacterial (Gabriela *et al.*, 2009, Palekar *et al.*, 2009), anti­viral (Upmanyu *et al.*, 2006), anti­microbial agents (Badr *et al.*, 2011) and anti­mycotic activity such as fluconazole, intra­conazole and voriconazole (Haber *et al.*, 2001). The search for new agent is one of the most challenging tasks to a medicinal chemist. The synthesis of high nitro­gen containing heterocyclic systems has been attracting increasing inter­est over the past decade because of their utility in various applications. In recent years, the chemistry of triazoles and their fused heterocyclic derivatives has received considerable attention owing to their synthetic and effective biological importance. The presence of three nitro­gen hetero-atoms in five membered ring system defines an inter­esting class of compounds. Keeping this in mind, we synthesized the title compound to study its crystal structure.

### 2.1. Synthesis and crystallization

To a stirred solution of 4-bromo-1-iodo-2-(tri­fluoro­meth­oxy)­benzene (1 g, 2.73mmol) in N,N-Di­methyl Formamide (10 mL), was added potassium carbonate ( 0.41g , 3.0 mmol), followed by 3-methyl-1H-1,2,4-triazole (0.23g 2.73 mmol). The mixture was stirred at room temperature for 30 minutes. Completion of the reaction was monitored by TLC. The reaction mixture was poured to 100g of crushed ice and the separated solid was filtered off and dried under vaccum.

Single crystals of the title compound were obtained from hexane-ethyl acetate (1:1 v/v) solvent system.

### 2.2. Refinement

Crystal data, data collection and structure refinement details are summarized in Table 1.

### 3. Results and discussion

In the title compound, C_10_H_7_BrF_3_N_3_O, the dihedral angle between the two planes defined by the benzene ring and the triazole ring is 23.17 (12)°. In the crystal structure, the molecules are linked to one another through C2—H2···N3 inter­actions generating zig zag C(7) chains running along [010].

### 4. Refinement

The H atoms were positioned with idealized geometry using a riding model with C—H = 0.93–0.96 Å. The isotropic displacement parameters for all H atoms were set to 1.2–1.5 times *U*_eq_ (Carbon).

### Crystal data

**Table:**

C_10_H_7_BrF_3_N_3_O	Prism
*M**_r_* = 322.10	*D*_x_ = 1.804 Mg m^−^^3^
Monoclinic, *P*2_1_/*n*	Melting point: 453 K
Hall symbol: -P 2yn	Mo *K*α radiation, λ = 0.71073 Å
*a* = 5.2389 (3) Å	Cell parameters from 25 reflections
*b* = 16.1548 (8) Å	θ = 1.9–30.1°
*c* = 14.0315 (7) Å	µ = 3.50 mm^−^^1^
β = 92.673 (3)°	*T* = 293 K
*V* = 1186.24 (11) Å^3^	Prism, colourless
*Z* = 4	0.33 × 0.21 × 0.14 mm
*F*(000) = 632	

### Data collection

**Table:**

Bruker APEXII CCD diffractometer	3499 independent reflections
Radiation source: fine-focus sealed tube	2373 reflections with *I* > 2σ(*I*)
Graphite monochromator	*R*_int_ = 0.051
ω scans	θ_max_ = 30.1°, θ_min_ = 1.9°
Absorption correction: multi-scan (*SADABS*; Bruker, 2009)	*h* = −7→7
*T*_min_ = 0.419, *T*_max_ = 0.613	*k* = −22→22
37092 measured reflections	*l* = −19→19

### Refinement

**Table:**

Refinement on *F*^2^	Secondary atom site location: difference Fourier map
Least-squares matrix: full	Hydrogen site location: inferred from neighbouring sites
*R*[*F*^2^ > 2σ(*F*^2^)] = 0.038	H-atom parameters constrained
*wR*(*F*^2^) = 0.135	*w* = 1/[σ^2^(*F*_o_^2^) + (0.0871*P*)^2^ + 0.1014*P*] where *P* = (*F*_o_^2^ + 2*F*_c_^2^)/3
*S* = 0.99	(Δ/σ)_max_ = 0.001
3499 reflections	Δρ_max_ = 0.41 e Å^−^^3^
165 parameters	Δρ_min_ = −0.68 e Å^−^^3^
0 restraints	Extinction correction: *SHELXL97* (Sheldrick, 2008), Fc^*^=kFc[1+0.001xFc^2^λ^3^/sin(2θ)]^-1/4^
Primary atom site location: structure-invariant direct methods	Extinction coefficient: 0.0045 (16)

### Special details

**Table:**

Geometry. All s.u.'s (except the s.u. in the dihedral angle between two l.s. planes) are estimated using the full covariance matrix. The cell s.u.'s are taken into account individually in the estimation of s.u.'s in distances, angles and torsion angles; correlations between s.u.'s in cell parameters are only used when they are defined by crystal symmetry. An approximate (isotropic) treatment of cell s.u.'s is used for estimating s.u.'s involving l.s. planes.
Refinement. Refinement of *F*^2^ against ALL reflections. The weighted *R*-factor *wR* and goodness of fit *S* are based on *F*^2^, conventional *R*-factors *R* are based on *F*, with *F* set to zero for negative *F*^2^. The threshold expression of *F*^2^ > 2σ(*F*^2^) is used only for calculating *R*-factors(gt) *etc*. and is not relevant to the choice of reflections for refinement. *R*-factors based on *F*^2^ are statistically about twice as large as those based on *F*, and *R*- factors based on ALL data will be even larger.

### Fractional atomic coordinates and isotropic or equivalent isotropic displacement parameters (Å^2^)

**Table:**

	*x*	*y*	*z*	*U*_iso_*/*U*_eq_	
C1	−0.1456 (5)	0.66662 (15)	0.14761 (15)	0.0523 (5)	
C2	0.0451 (4)	0.63544 (14)	0.20903 (15)	0.0524 (5)	
H2	0.1392	0.6703	0.2500	0.063*	
C3	0.0922 (4)	0.55182 (13)	0.20808 (14)	0.0453 (4)	
C4	−0.0376 (4)	0.49886 (12)	0.14480 (14)	0.0427 (4)	
C5	−0.2245 (4)	0.53249 (14)	0.08273 (16)	0.0524 (5)	
H5	−0.3122	0.4982	0.0392	0.063*	
C6	−0.2820 (5)	0.61578 (15)	0.08462 (17)	0.0559 (5)	
H6	−0.4107	0.6374	0.0441	0.067*	
N1	0.0066 (3)	0.41252 (11)	0.14056 (12)	0.0446 (4)	
C7	0.2131 (4)	0.36565 (15)	0.16360 (18)	0.0541 (5)	
H7	0.3662	0.3859	0.1905	0.065*	
C8	−0.0743 (4)	0.28860 (13)	0.10549 (17)	0.0508 (5)	
C9	−0.2132 (6)	0.21249 (15)	0.0729 (3)	0.0708 (8)	
H9A	−0.3924	0.2244	0.0646	0.106*	
H9B	−0.1874	0.1697	0.1198	0.106*	
H9C	−0.1494	0.1944	0.0134	0.106*	
C10	0.2286 (6)	0.50572 (18)	0.36008 (19)	0.0688 (7)	
N2	−0.1818 (3)	0.36208 (11)	0.10201 (14)	0.0492 (4)	
N3	0.1693 (4)	0.28792 (12)	0.14296 (18)	0.0582 (5)	
O1	0.2873 (3)	0.51928 (11)	0.26983 (13)	0.0592 (4)	
F1	0.0294 (6)	0.45837 (17)	0.36571 (16)	0.1342 (11)	
F2	0.4236 (6)	0.46877 (15)	0.40292 (16)	0.1310 (10)	
F3	0.1867 (5)	0.57307 (12)	0.40782 (12)	0.0998 (6)	
Br1	−0.22139 (7)	0.780974 (16)	0.14867 (2)	0.07657 (17)	

### Atomic displacement parameters (Å^2^)

**Table:**

	*U*^11^	*U*^22^	*U*^33^	*U*^12^	*U*^13^	*U*^23^
C1	0.0645 (12)	0.0470 (11)	0.0453 (11)	0.0065 (9)	0.0010 (9)	0.0023 (8)
C2	0.0605 (12)	0.0480 (11)	0.0478 (11)	−0.0036 (9)	−0.0062 (9)	−0.0017 (8)
C3	0.0458 (10)	0.0467 (10)	0.0425 (10)	0.0017 (8)	−0.0066 (8)	0.0012 (8)
C4	0.0415 (9)	0.0416 (10)	0.0447 (10)	0.0022 (7)	−0.0012 (7)	−0.0007 (8)
C5	0.0536 (11)	0.0507 (11)	0.0514 (12)	0.0059 (9)	−0.0128 (9)	−0.0030 (9)
C6	0.0634 (13)	0.0511 (12)	0.0520 (12)	0.0118 (10)	−0.0099 (10)	0.0037 (9)
N1	0.0415 (8)	0.0438 (9)	0.0479 (9)	0.0036 (6)	−0.0040 (6)	−0.0028 (7)
C7	0.0411 (10)	0.0539 (12)	0.0664 (14)	0.0093 (9)	−0.0077 (9)	−0.0052 (10)
C8	0.0507 (11)	0.0471 (12)	0.0547 (12)	0.0030 (8)	0.0024 (9)	−0.0046 (9)
C9	0.0684 (16)	0.0487 (14)	0.095 (2)	−0.0032 (10)	0.0046 (14)	−0.0127 (12)
C10	0.0948 (19)	0.0510 (13)	0.0577 (15)	0.0047 (12)	−0.0265 (13)	0.0011 (10)
N2	0.0410 (8)	0.0462 (9)	0.0598 (10)	−0.0013 (7)	−0.0046 (7)	−0.0023 (8)
N3	0.0511 (10)	0.0518 (11)	0.0713 (14)	0.0114 (8)	−0.0016 (9)	−0.0039 (8)
O1	0.0567 (8)	0.0600 (9)	0.0589 (9)	0.0055 (7)	−0.0205 (7)	−0.0029 (7)
F1	0.181 (2)	0.139 (2)	0.0812 (14)	−0.080 (2)	−0.0096 (15)	0.0274 (13)
F2	0.177 (2)	0.1229 (19)	0.0866 (14)	0.0713 (17)	−0.0627 (15)	−0.0062 (13)
F3	0.1628 (19)	0.0694 (11)	0.0658 (10)	0.0264 (12)	−0.0112 (11)	−0.0113 (8)
Br1	0.1162 (3)	0.04531 (19)	0.0671 (2)	0.01567 (12)	−0.00788 (17)	0.00117 (10)

### Geometric parameters (Å, º)

**Table:**

C1—C6	1.381 (3)	N1—N2	1.372 (2)
C1—C2	1.383 (3)	C7—N3	1.307 (3)
C1—Br1	1.890 (2)	C7—H7	0.9300
C2—C3	1.373 (3)	C8—N2	1.314 (3)
C2—H2	0.9300	C8—N3	1.358 (3)
C3—C4	1.388 (3)	C8—C9	1.490 (3)
C3—O1	1.410 (2)	C9—H9A	0.9600
C4—C5	1.390 (3)	C9—H9B	0.9600
C4—N1	1.416 (3)	C9—H9C	0.9600
C5—C6	1.379 (3)	C10—F1	1.299 (4)
C5—H5	0.9300	C10—F2	1.306 (3)
C6—H6	0.9300	C10—F3	1.302 (3)
N1—C7	1.347 (3)	C10—O1	1.335 (3)
			
C6—C1—C2	121.3 (2)	N3—C7—N1	110.9 (2)
C6—C1—Br1	118.93 (17)	N3—C7—H7	124.6
C2—C1—Br1	119.80 (17)	N1—C7—H7	124.6
C3—C2—C1	118.5 (2)	N2—C8—N3	114.54 (19)
C3—C2—H2	120.8	N2—C8—C9	122.1 (2)
C1—C2—H2	120.8	N3—C8—C9	123.3 (2)
C2—C3—C4	121.97 (18)	C8—C9—H9A	109.5
C2—C3—O1	119.08 (18)	C8—C9—H9B	109.5
C4—C3—O1	118.86 (18)	H9A—C9—H9B	109.5
C5—C4—C3	118.01 (19)	C8—C9—H9C	109.5
C5—C4—N1	118.07 (18)	H9A—C9—H9C	109.5
C3—C4—N1	123.91 (18)	H9B—C9—H9C	109.5
C4—C5—C6	121.1 (2)	F1—C10—F2	108.4 (3)
C4—C5—H5	119.4	F1—C10—F3	107.8 (3)
C6—C5—H5	119.4	F2—C10—F3	107.0 (2)
C1—C6—C5	119.0 (2)	F1—C10—O1	112.0 (2)
C1—C6—H6	120.5	F2—C10—O1	107.6 (3)
C5—C6—H6	120.5	F3—C10—O1	113.8 (2)
C7—N1—N2	108.42 (17)	C8—N2—N1	102.87 (16)
C7—N1—C4	132.43 (18)	C7—N3—C8	103.32 (18)
N2—N1—C4	119.06 (16)	C10—O1—C3	116.85 (18)
			
C6—C1—C2—C3	−1.7 (3)	C3—C4—N1—N2	157.37 (19)
Br1—C1—C2—C3	178.63 (17)	N2—N1—C7—N3	−0.5 (3)
C1—C2—C3—C4	2.8 (3)	C4—N1—C7—N3	−176.8 (2)
C1—C2—C3—O1	179.54 (19)	N3—C8—N2—N1	−0.3 (3)
C2—C3—C4—C5	−1.6 (3)	C9—C8—N2—N1	178.3 (2)
O1—C3—C4—C5	−178.29 (19)	C7—N1—N2—C8	0.4 (2)
C2—C3—C4—N1	179.3 (2)	C4—N1—N2—C8	177.29 (19)
O1—C3—C4—N1	2.6 (3)	N1—C7—N3—C8	0.3 (3)
C3—C4—C5—C6	−0.8 (3)	N2—C8—N3—C7	0.0 (3)
N1—C4—C5—C6	178.4 (2)	C9—C8—N3—C7	−178.5 (3)
C2—C1—C6—C5	−0.6 (4)	F1—C10—O1—C3	55.1 (3)
Br1—C1—C6—C5	179.06 (18)	F2—C10—O1—C3	174.1 (2)
C4—C5—C6—C1	1.9 (4)	F3—C10—O1—C3	−67.6 (3)
C5—C4—N1—C7	154.2 (2)	C2—C3—O1—C10	81.4 (3)
C3—C4—N1—C7	−26.6 (3)	C4—C3—O1—C10	−101.8 (2)
C5—C4—N1—N2	−21.8 (3)		

### Hydrogen-bond geometry (Å, º)

**Table:**

*D*—H···*A*	*D*—H	H···*A*	*D*···*A*	*D*—H···*A*
C2—H2···N3^i^	0.93	2.59	3.511 (3)	169

Symmetry code: (i) −*x*+1/2, *y*+1/2, −*z*+1/2.
